# Evaluation of the antioxidant profile and cytotoxic activity of red propolis extracts from different regions of northeastern Brazil obtained by conventional and ultrasound-assisted extraction

**DOI:** 10.1371/journal.pone.0219063

**Published:** 2019-07-05

**Authors:** João Henrique de Oliveira Reis, Gabriele de Abreu Barreto, Jamile Costa Cerqueira, Jeancarlo Pereira dos Anjos, Luciana Nalone Andrade, Francine Ferreira Padilha, Janice Izabel Druzian, Bruna Aparecida Souza Machado

**Affiliations:** 1 Federal University of Bahia, Faculty of Pharmacy, Salvador, Bahia, Brazil; 2 University Center SENAI/CIMATEC, National Service of Industrial Learning – SENAI, Heath Institute of Technology (ITS CIMATEC), Salvador, Bahia, Brazil; 3 Federal University of Sergipe, Lagarto, Sergipe, Brazil; 4 Institute of Research and Technology (ITP), Tiradentes University, Aracaju, Sergipe, Brazil; Tallinn University of Technology, ESTONIA

## Abstract

Propolis is a complex mixture of resinous and balsamic material collected from the exudates of plants, shoots, and leaves by bees. This study evaluated red propolis extracts obtained by conventional (ethanolic) extraction and ultrasound-assisted extraction of six samples from different regions of northeastern Brazil. The total phenolic compounds and flavonoids, *in vitro* antioxidant activity, concentration of formononetin and kaempferol and the cytotoxicity against four human tumor cell lines were determined for all twelve obtained extracts. Significant variations in the levels of the investigated compounds were identified in the red propolis extracts, confirming that the chemical composition varied according to the sampling region. The extraction method used also influenced the resulting propolis compounds. The highest concentration of the compounds of interest and the highest *in vitro* antioxidant activity were exhibited by the extracts obtained from samples from state of Alagoas. Formononetin and kaempferol were identified in all samples. The highest formononetin concentrations were identified in extracts obtained by ultrasound, thus indicating a greater selectivity for the extraction of this compound by this method. Regarding cytotoxic activity, for the HCT-116 line, all of the extracts showed an inhibition of greater than 90%, whereas for the HL-60 and PC3 lines, the minimum identified was 80%. In general, there was no significant difference (p>0.05) in the antiproliferative potential when comparing the extraction methods. The results showed that the composition of Brazilian red propolis varies significantly depending on the geographical origin and that the method used influences the resulting compounds that are present in propolis. However, regardless of the geographical origin and the extraction method used, all the red propolis samples studied presented great biological potential and high antioxidant activity. Furthermore, the ultrasound-assisted method can be efficiently applied to obtain extracts of red propolis more quickly and with high concentration of biomarkers of interest.

## Introduction

Propolis is a complex mixture formed by resinous and balsamic material originating from various parts of plants, such as shoots, exudates, branches, and leaves, that is collected by different bee species [1–3]. It is used by bees to protect the beehive against insects and to prevent the proliferation of invading microorganisms, thus functioning as a protective barrier [4–6]. In general, propolis is composed of around 50% resins and plant balsams, 30% wax, 10% essential oils, 5% pollen and 5% of other substances and materials, including organic compounds [7–9]. More than 300 chemical compounds of interest have already been identified in propolis samples of different geographical origins, [10,11] and the major constituents of propolis are phenolic compounds, which have been extensively studied to date as antioxidants present in natural products [12–14]. Therefore, different kinds of propolis are present all over the world and each propolis is chemically different and has specific properties and applications [15,16].

Brazilian red propolis is primarily found in the coastal region of northeastern Brazil, and its chemical composition is highly variable and directly related to the compounds found in its main botanical origin, *Dalbergia ecastaphyllum* (L) Taub. [2,17–21]; however, a second plant species likely participates as one of the main sources of resins for red propolis [11]. Presently, it is the second most produced and traded type of Brazilian propolis, being produced mainly on the littoral of the state of Alagoas (northeast Brazil) [22]. The biological activity of red propolis is mainly due to isoflavones, which act in synergy with the other compounds. Formononetin is the main isoflavone present in red propolis samples [23,24]. Other compounds identified in the fractions and extracts from Brazilian red propolis, such as vestitol, neovestitol, biochanin A and liquiritigenina, are also considered important markers and have been associated with different biological effects [25,26].

Different studies have demonstrated a wide variety of biological activities for red propolis extracts, such as antioxidant [2,19], antimicrobial [27,28], antitumor [1,29,30], anti-inflammatory [31,32], antiparasitic [33–35], and anti-nociceptive activities [32]. Red propolis is currently recognized as the most promising type of propolis because of its biotechnological potential. The phenolic compounds, including the flavonoids, have been considered the main biologically active constituents of this resin, together with the cinnamic acid derivatives, esters, and some terpenes [3,25].

The chemical composition of propolis, and consequently its biological activity, varies according to its geographical origin, botanical source, race of bees, sampling season and climate conditions of the region [36–40]. As a result, different studies have investigated the influence of different factors on the chemical composition of propolis [41–46]. However, few available studies have compared red propolis samples collected in different regions of Brazil regarding their antioxidant composition and cytotoxic activity against different tumor cell lines. Studies evaluating the biological activities of red propolis performed by Machado et al. [27], Silva et al. [1], and Teles et al. [47] found differences in antimicrobial and antitumor capacity, antioxidant and antiparasitic capacity, and hypertension and renal damage attenuation capacity, respectively, for samples obtained from different sources. Regueira-Neto et al. [28] investigated the effect of seasonality on the antibacterial activity and chemical composition of a Brazilian red propolis sample and found an important variation in the concentrations of the investigated compounds and, consequently, in the antibacterial activity of the extracts according to the sampling period (dry vs. rainy season).

Several methods are used worldwide to extract the propolis components, and extraction using ethanol as a solvent is the most commonly used method [48]. Ethanolic extracts have been more commonly used due to of their content in phenolic acids and flavonoids [49]. Different studies describe different chemical compositions and biological activities for propolis extracts depending on the extraction method employed, demonstrating that the extraction conditions, as well as the extraction solvent used, directly influence the yield and selectivity of some compounds [50–55] and, consequently, the biotechnological potential of the extracts obtained. Thus, although ethanol extraction is the method most commonly used by the industry to obtain different types of propolis extracts, this method has the disadvantages of low selectivity and low yield in the extraction of some compounds of interest in addition to long extraction periods [51], thus increasing the extraction costs. Therefore, other methods have been used to increase the efficiency of the extraction of the bioactive components of propolis, such as ultrasound and microwave-assisted extraction and supercritical fluid extraction [48,56–58].

In this context, ultrasound-assisted extraction represents a reliable alternative to traditional extraction methods and has been widely applied in the extraction of compounds from different natural matrices [59–62]. The study by Tan et al. [63] demonstrated greater avocado oil extraction efficiency with ultrasound-assisted extraction and supercritical fluid extraction when compared to conventional methods. Figueiredo et al. [64] demonstrated the higher efficiency of ultrasound-assisted extraction to obtain phytosterols in vegetable oils.

Despite the advantages of ultrasound technology to obtain compounds of interest in a shorter time with higher yields and lower solvent consumption when compared to the conventional extraction methods, few studies have investigated the extraction of propolis extracts using this technology [65–67]. Thus, the aim of this study was to evaluate the antioxidant profile and *in vitro* cytotoxic activity of extracts obtained by conventional extraction and ultrasound-assisted extraction of six red propolis samples collected in different regions of northeastern Brazil.

## Materials and methods

### Materials

Ethanol, methanol, acetic acid, aluminum chloride, DMSO (dimethyl sulfoxide), Folin-Ciocalteu reagent and the standards kaempferol (CAS number 520-18-3), rutin hydrate (CAS Number 207671-50-9), formononetin (CAS number 485-72-3), gallic acid (CAS number 149-91-7), quercetin (CAS number 117-39-5), p-coumaric acid (CAS number 501-98-4), epicatechin (CAS number 490-46-0), caffeic acid (CAS number 331-39-5), catechin (CAS number 7295-85-4), 2,2-diphenyl-1-picrylhydrazyl (DPPH) (CAS number 1898-66-4), and trans-ferulic acid (CAS number 537-98-4) were purchased from Sigma-Aldrich Chemical Co. (St. Louis, MO, USA). A 0.45-μm regenerated cellulose membrane filter (SLCR025NS, Millipore Corporation Co., Bedford, Mass., USA) was used.

### Obtaining and processing raw red propolis from northeastern Brazil

**Fig 1:**
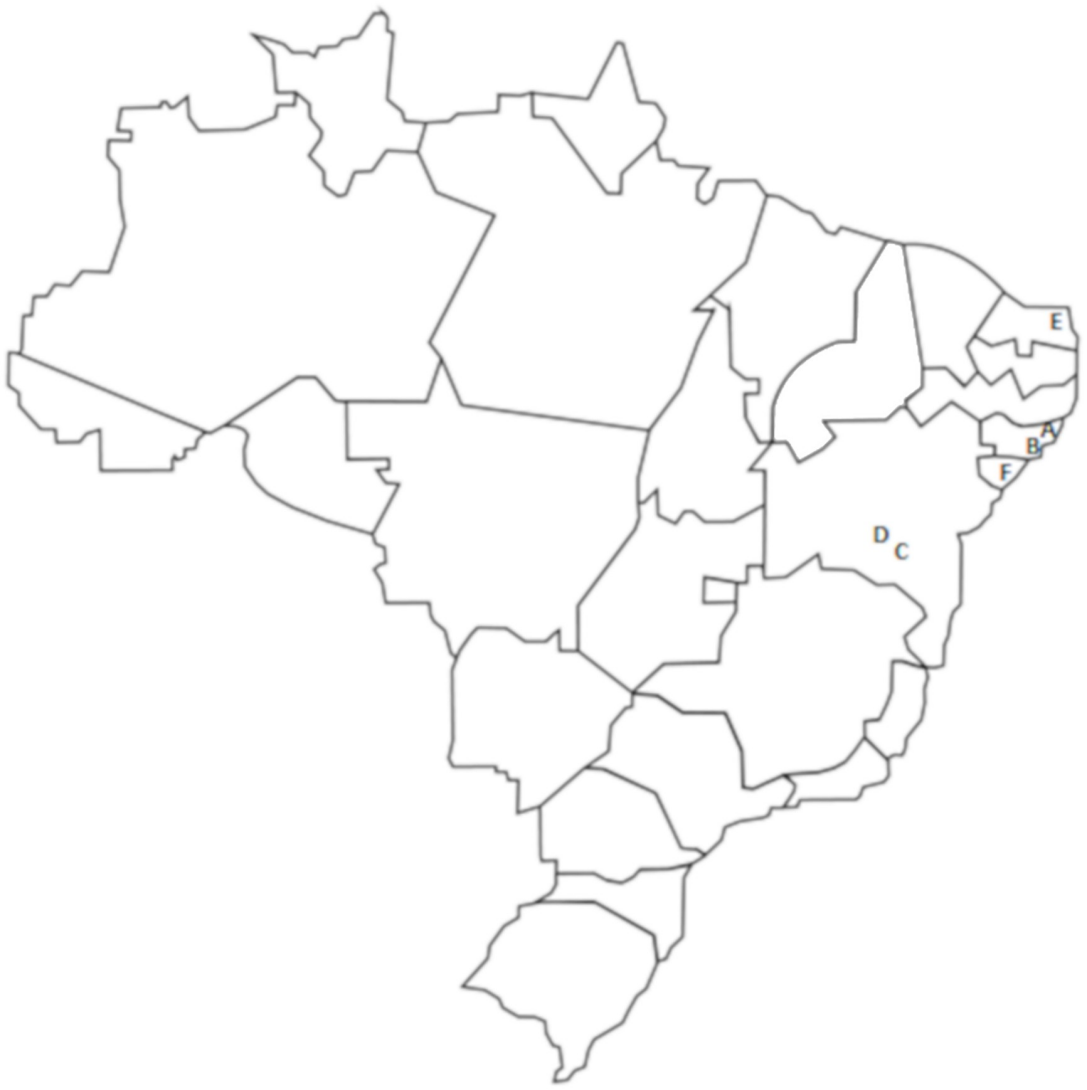
Approximate geographical location of the samples of the red propolis evaluated (A and B—Alagoas; C and D—Bahia; E—Rio Grande do Norte; and, F—Sergipe).

Approximately 800 g of each red propolis sample were obtained from six different apiaries located in northeastern Brazil (Fig 1), more specifically in the states of Alagoas (samples A and B), Bahia (samples C and D), Rio Grande do Norte (sample E) and Sergipe (sample F), as shown in Table 1. The different samples were donated by the companies Apis Jordans (Vitória da Conquista—Bahia—Brazil), Apis Nativa Produtos Naturais (Prodapys—Santa Catarina—Brazil) and Bee Product Natural (Alagoas—Brazil). The samples were ground in an electric mill (Cadence—Brazil) and sieved through a 52–92 μm aluminum sieve for uniformity of particle size and to increase the surface area. The samples were stored in an ultra-freezer at -20°C and were protected from light in an inert atmosphere (N_2_) to avoid degradation of the material.

**Table 1:** Identification, extraction method used and geographic location of red propolis samples from different regions of northeastern Brazil.

Sample identification	State of Brazil	Extraction method	Geographic location
A1	Alagoas	Conventional	9°41'59.7"S, 36°20'10.7"W
A2	Alagoas	Ultrasound-assisted	9°41'59.7"S, 36°20'10.7"W
B1	Alagoas	Conventional	9°46'10.9"S 35°50'52.4"W
B2	Alagoas	Ultrasound-assisted	9°46'10.9"S 35°50'52.4"W
C1	Bahia	Conventional	12°53'13.9"S, 40°56'38.5"W
C2	Bahia	Ultrasound-assisted	12°53'13.9"S, 40°56'38.5"W
D1	Bahia	Conventional	15°40'24.4"S 38°56'42.8"W
D2	Bahia	Ultrasound-assisted	15°40'24.4"S 38°56'42.8"W
E1	Rio Grande do Norte	Conventional	5°39'52.8"S, 36°15'35.8"W
E2	Rio Grande do Norte	Ultrasound-assisted	5°39'52.8"S, 36°15'35.8"W
F1	Sergipe	Conventional	10°28'10.5"S, 37°17'47.3"W
F2	Sergipe	Ultrasound-assisted	10°28'10.5"S, 37°17'47.3"W

In this study we used six samples of red propolis collected in different regions of Brazil and two extraction methods were used to obtain the extracts, totaling 12 extracts. Number 1 after the letter means the conventional method of extraction and the number 2 after the letter means the method with the application of the ultrasound.

A1, B1, C1, D1, E1 and F1—Extracts obtained by conventional extraction; A2, B2, C2, D2, E2 and F2—Extracts obtained by ultrasound-assisted extraction.

### Obtaining the extracts

The extracts from the six red propolis samples were obtained by two methods: conventional extraction (A1, B1, C1, D1, E1, and F1) and ultrasound-assisted extraction (A2, B2, C2, D2, E2, and F2), totaling 12 extracts (Table 1).

For the ultrasound-assisted extraction, the methodology of Chen et al. [68] was used with modifications. Thus, 2 g of each propolis sample was homogenized with 25 mL of ethanol:water (80:20 v/v) in an Erlenmeyer flask and placed in an ultrasonic bath (RMS, Quimis, Brazil) with a power of 200  W, frequency of 60  kHz, and a temperature of 50°C for 50 minutes. Conventional extraction was carried out in a similar way to the industrial process used for propolis extracts in Brazil. The same amount of each red propolis sample (2 g) was infused with ethanol:water (80:20 v/v) and allowed to stand for seven days with periodic shaking (25°C). During the extraction process (conventional or ultrasound-assisted) all samples were kept protected from light.

Next, the extracts obtained by the two methods were centrifuged in a refrigerated centrifuge (Routine 380R, Hettich, Germany) at 20,000 RPM for 10 minutes at 4°C, and the resulting supernatant was filtered on qualitative filter paper (80 g). Finally, the extracts were dried at 40°C in a forced-air oven (Thermo Scientific, Massachusetts, USA) until reaching constant weight.

### Identification and quantification of compounds by HPLC

The quantification and identification of ten phenolic compounds (caffeic acid, gallic acid, formononetin, kaempferol, trans-ferulic acid, *p*-coumaric acid, catechin, epicatechin, quercetin, and rutin hydrate) in the red propolis extracts was performed by high-performance liquid chromatography (HPLC). Initially, solutions of 10 mg.mL^-1^ were prepared and dissolved in methanol and then placed in an ultrasonic bath (TECNAL, São Paulo, Brazil) for 30 minutes. Methanol solutions of the red propolis extracts were prepared at 1 mg.mL^-1^ with the two methods adopted in this study. The samples were filtered with a 0.45 μm cellulose membrane filter (Millipore) for subsequent injection into an HPLC system (Shimadzu, LC-20AT, Japan) equipped with an automatic injector and diode array detector (DAD) (Shimadzu, SPD-M20, Japan). The chromatographic separation was performed according to the methodology proposed by Castro et al. [69] and Cabral et al. [70]. A NUCLEODUR 100–5 C18 EC column (150 x 4 mm ID, 5-μm particle size) was used in conjunction with a ZORBAX Eclipse Plus C18 4.6 x 12.5 mm precolumn (Agilent, USA).

Gradient elution, with a mobile phase of 5% acetic acid and methanol at different ratios and with a total analysis time of 42 minutes (from 0 to 35 minutes (0–92% B), 35 to 40 minutes (92–0% B), and 40 to 42 minutes (0% B)) was used as the analysis condition. The injection volume was 20 μL, and the flow rate was 1 mL.min^-1^. The machine was operated at a temperature of 25±2°C. The detection wavelengths were set at 300 and 320 nm, and the DAD was operated within a wavelength range of 190 to 800 nm. For the identification of the compounds, comparisons of retention time and ultraviolet spectrum were performed between samples and standards. This analysis was performed according to the parameters of limits of detection, and limits of quantification [71,72] (Table 2).

**Table 2:** HPLC identification and quantification parameters of phenolic compounds from six red propolis samples obtained by conventional and ultrasound-assisted extraction.

Standards	t_R_ (min)	ʎ (nm)	Working range (mg.L^-1^)	LD (mg.L^-1^)	LQ (mg.L^-1^)
Gallic acid	2.26	280	1.0–12.5	0.92	3.05
Caffeic acid	8.13	30	1.0–15.0	0.82	2.73
Trans-ferulic acid	11.38	320	0.5–12.5	0.28	0.92
*p*-Coumaric acid	10.36	300	1.0–15.0	0.82	2.72
Catechin	6.42	280	1.0–15.0	0.81	2.68
Epicatechin	8.44	280	0.5–15.0	0.28	0.93
Formononetin	19.46	300	0.5–12.5	0.31	1.02
Kaempferol	17.53	320	0.5–12.5	0.12	0.41
Quercetin	15.30	320	0.5–12.5	0.21	0.71
Rutin hydrate	11.00	320	0.5–12.5	0.27	0.91

TR = retention time, ʎ = wavelength, LD = limit of detection and LQ = limit of quantification of the ten phenolic compounds investigated in the samples studied by HPLC.

### Content of total phenolic compounds by spectrophotometry

The content of total phenolic compounds in the red propolis extracts obtained by the two extraction methods was determined using the methodology of Singleton et al. [73] and Singleton et al. [74], which are based on the reaction with the Folin-Ciocalteau reagent. First, the reaction was prepared with a 0.5-mL aliquot of each propolis extract dissolved in 95% ethanol to a final concentration of 500 μg.mL^-1^, 10% aqueous Folin-Ciocalteu solution (2.5 mL) and 7.5% sodium carbonate (2.0 mL). The vials containing the obtained mixture were heated in a temperature-controlled bath at 50°C for 5 minutes (Marconi, M127, Brazil), after which the absorbance was read in a UV/Vis spectrophotometer (PerkinElmer, LAMBDA 25 UV/Vis Systems, WA, USA) at 765 nm using a quartz cuvette with a 10 mm optical path and a 3.5 mL volume. The amount of total phenolic compounds was expressed as Gallic acid equivalents per gram of sample (mgGAE.g^-1^) by calculating a calibration curve (y = 0.0096x–0.0311, R^2^ = 0.9994) using Gallic acid standard solutions (12 to 200 μg.mL^-1^) under the same conditions.

### Content of total flavonoid compounds by spectrophotometry

The content of total flavonoid compounds was determined using the method proposed by Meda et al. [75] with adaptations. First, 2.0 mL of each extract (0.5 mg.mL^-1^) was added into test tubes along with 2.0 mL of a 2% methanol solution of aluminum chloride (AlCl_3_). The samples were then homogenized on a vortex shaker (IKA Lab Dancer, Germany) and placed in the dark for 30 minutes, after which the absorbance was read in a UV/Vis spectrophotometer (PerkinElmer, LAMBDA 25 UV/Vis System) at the wavelength of 415 nm. A quercetin standard curve (5 to 105 μg.mL^-1^) was obtained under the same conditions (y = 0.0271x–0.014, R^2^ = 0.9994), and the amount of total flavonoids in the extracts was expressed as quercetin equivalents per gram of sample (mgQE.g^-1^).

### DPPH (2,2-diphenyl-1-picrylhydrazine): *In vitro* antioxidant activity

To determine antioxidant capacity, the 2,2-diphenyl-1-picrylhydrazine reactive (DPPH) method was used according to the methodologies proposed by Brand-Williams et al. [76] and Molyneux et al. [77] with adaptations. First, six dilutions of each extract were prepared at concentrations of 10 to 85 μg.mL^-1^ (in triplicate). Next, a 1-mL aliquot of each dilution was transferred to test tubes containing 3.0 mL of ethanol solution (99%) of the DPPH● radical (0.004%). The DPPH free radical reduction was determined by reading the absorbance at a wavelength of 517 nm (calibration curve y = 0.897x–4.5, R2 = 0.9955) with a UV/Vis spectrophotometer (PerkinElmer, LAMBDA 25 UV/Vis System) after 30 minutes of incubation in the dark at 25°C.

The free radical scavenging capacity was expressed as the percentage inhibition of the radical oxidation and calculated according to Eq 1. A similar procedure was used for the blank, where the extract sample was replaced with ethanol. The EC_50_ value (effective concentration of extract required to scavenge DPPH● radical by 50%) was obtained and was based on the line equation for the extract concentrations and respective percentages of DPPH● radical scavenging.

%scavenging=100−[(finalabsorbanceofsamplex100)/absorbanceoftheblank]. (1)

### *In vitro* cytotoxic activity

The human tumor cell lines HL-60 (leukemia), PC3 (prostate carcinoma), SNB19 (glioblastoma), and HCT-116 (colon carcinoma) were kindly provided by the National Cancer Institute (USA) and used for the analysis of *in vitro* cytotoxicity of the different Brazilian red propolis extracts. All cell lines were cultured in RPMI 1640 complete medium (Gibco, Life Technologies, Carlsbad, CA, USA) with 10% fetal bovine serum (FBS) (Gibco) and 1% penicillin/streptomycin antibiotic solution and were incubated in an incubator (Thermo Scientific, 3425, Massachusetts, USA) at 37°C with 5% CO_2_. Trypsin (0.25%) was used to detach the cells from the walls of the culture flasks.

The MTT [3-(4,5-dimethyl-2-thiazolyl) -2,5-diphenyl -2H- tetrazolium bromide] (Sigma Aldrich, Missouri, USA) assay was used for determining the cytotoxic (antitumor) potential of the extracts against the cell lines [78,79]. The samples were distributed in 96-well plates (100 μL.well^-1^) at a final concentration of 0.1x10^6^ cells.mL^-1^. After 24 hours, the extracts were dissolved in 0.001% DMSO and added to the wells to a final concentration of 50 μg.mL^-1^. The experiment was performed three independent times (in triplicate), with 0.25 μg.mL^-1^ doxorubicin and 0.001% DMSO as positive and negative controls, respectively (incubation for 72 hours in an incubator with 5% CO_2_, at 37°C). At the end of the incubation, the plates were centrifuged (15 g/15 min) at 4°C and the supernatants were discarded. Subsequently, 150 μL of the MTT solution (0.5 μg.mL^-1^) was added, and the plates were incubated for 3 hours. After this period, the plates were centrifuged again (3 g.min^-1^) at 4°C, the supernatants were discarded, and the precipitates were resuspended in 150 μL of sterile pure DMSO. For the quantification of formazan produced by viable cells, the absorbance was read using a multiplate reader (DTX 880 Multimode Detector, Beckman Coulter, Packard, ON, Canada) at a wavelength of 595 nm. All values were expressed as the 100% inhibitory concentration (IC100).

### Statistical analysis

The results of this study were expressed as the mean ± standard error of mean (SEM) (n = 3). The statistical analysis of the results was performed using the Statistica 6.0 software from StatSoft (Tulsa, USA). A one-way ANOVA and the Tukey test (95% confidence level) were used to identify the differences between the concentrations of phenolic compounds, flavonoids, concentration of compounds by HPLC, and antioxidant and cytotoxic activity in the extracts obtained through the two extraction methods and for the different propolis samples. In all statistical procedures, the level of significance was set at p<0.05.

## Results and discussion

### Antioxidant profile of red propolis extracts

**Fig 2:**
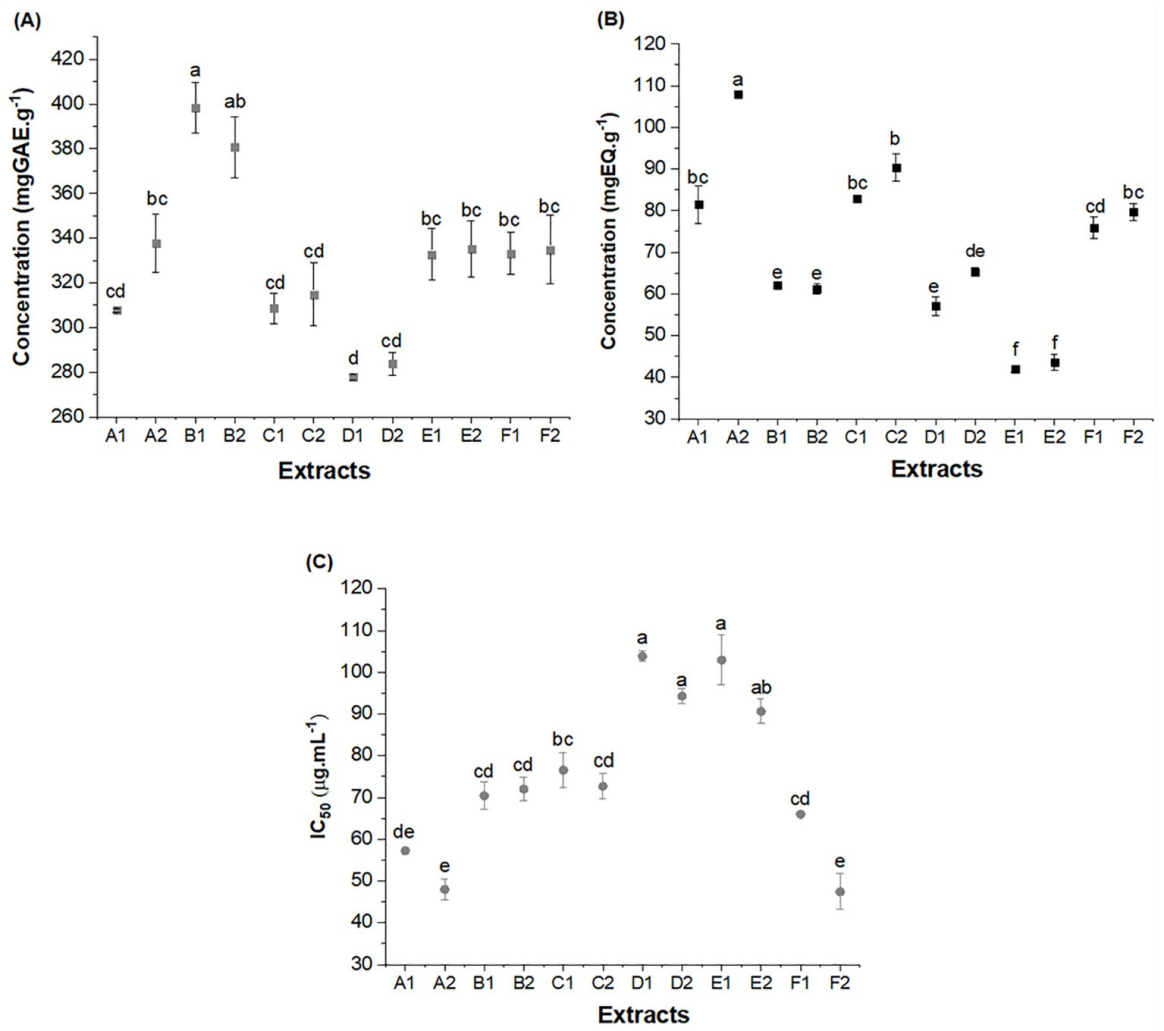
Total phenolic compound content expressed in mgGAE.g^-1^ (A); flavonoids expressed in mg mgQE.g^-1^ (B); and DPPH expressed as IC_50_ –μg.mL^-1^ (C) of the extracts of different samples of Brazilian red propolis (mean ± standard error of mean). IC_50_: Lower values of IC_50_ indicate higher activity of radical elimination. A1, B1, C1, D1, E1 and F1—Extracts obtained by conventional extraction; A2, B2, C2, D2, E2 and F2—Extracts obtained by ultrasound-assisted extraction. IC_50_: Lower values of IC_50_ indicate higher activity of radical elimination. Statistical analysis: Values showing the same letter in the same analysis do not show significant differences (p>0.05) based on the Tukey test at a 95% confidence level.

Table 3 and Fig 2 show the results for the total phenolic compounds, flavonoids, and antioxidant activity of the ethanol extracts of the different red propolis samples obtained by the two extraction methods (conventional and ultrasound-assisted).

**Table 3:** Determination of the content of total phenolic compounds (mgGAE.g^-1^), flavonoids (mgQE.g^-1^) and antioxidant activity (DPPH—IC_50_ μg.mL^-1^) of the extracts from Brazilian red propolis obtained by conventional (1) and ultrasound-assisted extraction (2) (mean ± standard error of mean).

Samples	Phenolic compounds (mgGAE.g^-1^)	Flavonoids (mgQE.g^-1^)	DPPH (IC_50_) (μg.mL^-1^)
A1	307.63±0.92^c.d^	81.42±4.45^b.c^	57.27±0.73^d.e^
A2	337.72±13.08^b.c^	108.02±0.18^a^	48.00±2.45^e^
B1	398.31±11.15^a^	62.01±0.51^e^	70.41±3.22^c.d^
B2	380.73±13.60^a.b^	61.17±1.18^e^	72.02±2.79^c.d^
C1	308.49±6.91^c.d^	82.87±0.35^b.c^	76.58±4.17^b.c^
C2	314.75±14.00^c.d^	90.38±3.36^b^	72.70±3.01^c.d^
D1	277.81±1.32^d^	57.07±2.20^e^	103.85±1.23^a^
D2	283.74±5.17^c.d^	65.34±0.85^d.e^	94.28±1.82^a^
E1	332.74±11.68^b.c^	42.00±0.75^d.f^	102.94±5.94^a^
E2	335.16±12.55^b.c^	43.64±1.90^f^	90.61±2.98^a.b^
F1	333.06±9.39^b.c^	75.89±2.50^c.d^	65.96±0.10^c.d^
F2	334.89±15.34^b.c^	79.67±2.10^b.c^	47.42±4.28^e^

The results of the quantitative analysis of phenolic compounds, flavonoids and DPPH in the extracts obtained by the two extraction methods from different Brazilian red propolis samples are presented. The samples A1, B1, C1, D1, E1 and F1 are the extracts obtained by conventional extraction and the samples A2, B2, C2, D2, E2 and F2 are the extracts obtained by ultrasound-assisted extraction. Lower values of IC_50_ indicate higher activity of radical elimination (DPPH results).

Statistical analysis: Values showing the same letter in the same column do not show significant difference (p>0.05) through the Tukey test at a 95% confidence level.

In general, a significant variation (p>0.05) was observed for phenolic compounds, flavonoids, and antioxidant activity among the extracts obtained for red propolis samples from different origins. The content of phenolic compounds ranged from 277.8±1.32 (D1) to 398.3±11.15 mgGAE.g^-1^ (B1), the flavonoid content from 42.0±0.75 (E1) to 108.0±0.18 mgQ.g^-1^ (A2), and the antioxidant activity from 102.94±5.94 (E1) to 47.42±4.28 (IC_50_) (F2) (Fig 2 and Table 2). The variations observed between the samples (p>0.05) were expected considering that the propolis obtained from different geographical regions exhibited different chemical profiles [4,27,28,45]. Samples of the same specific type of propolis (red color) show variation in the content of antioxidant compounds when collected in different geographic regions. Thus, the results found in this study confirm the effect of the origin of the raw material on the composition of the extracts.

The red propolis samples collected in the northeastern region of Brazil had high amounts of phenolic compounds and flavonoids, as well as a high antioxidant capacity, as previously demonstrated by Machado et al. [27] and Andrade et al. [66]. The phenolic compounds, specifically the flavonoids, are the main components responsible for the biological activity of propolis [33,80].

The highest amount of phenolic compounds was identified in sample B (extracts B1 and B2), while the highest flavonoid content and highest antioxidant activity were observed in sample A (extracts A1 and A2), both from the state of Alagoas. Notably, that the Brazilian red propolis produced in Alagoas is the only propolis in the country that has a certificate of origin (geographical indication) due to the scientific recognition of its differentiated chemical composition [81,82].

When comparing the results from other studies that also evaluated the antioxidant profile of Brazilian propolis, the current study found higher phenolic concentrations (approximately 4-fold, sample B) than those reported in the studies by Cottica al. [83] and Mello et al. [84], which found values ranging from 48 to 87 mgGAE.g^-1^ and 49 to 100 mgGAE.g^-1^, respectively.

In regards to the flavonoid content, the values obtained are in agreement with the literature for red propolis [18,85,86]. Righi et al. [2] reported a variation of between 27 and 43 mgQE.g^-1^, while Alencar et al. [19], Hatano et al. [87] and Wang et al. [88] obtained flavonoid concentrations ranging from 43 to 55 mgQE.g^-1^ when they evaluated different types of propolis.

Red propolis, regardless of its origin, has a high antioxidant potential, as has been demonstrated in previous studies [31,32,84,89]. Andrade et al. [66] showed a higher antioxidant capacity of red propolis when comparing samples of different types of propolis (green, red, and brown) from the Brazilian northeast.

In this study, sample A (from Alagoas) had the highest antioxidant activity, which was represented by the lower IC_50_ (extracts A1 and A2). These results indicate that the chemical nature of the phenolic compounds, and perhaps the presence of other compounds, contributes to the total antioxidant capacity of the extracts [51]. Similar results for antioxidant activity were identified by Alencar et al. [90] (57.0±3.2%) when evaluating the ethanol extract of red propolis from Alagoas. Frozza et al. [91] found an IC_50_ of 270.13±24.77 μg.mL^-1^ for ethanol extracts of red propolis from Sergipe state (Brazil). Machado et al. [27] observed IC_50_ values of between 31 and 183 μg.mL^-1^ in Brazilian red propolis extracts (Sergipe and Alagoas), while Christov et al. [92] found values of between 65 and 79% inhibition by DPPH for the ethanol extract of propolis from Canada at 210 μg.mL^-1^.

When evaluating the extracts obtained by the different methods (conventional and ultrasound-assisted) from the same sample, in general, no significant differences were identified (p>0.05) (Table 3 and Fig 2). However, according to Dent et al. [93] ultrasound-assisted extraction is the rapid extraction technique, which in comparison to conventional extraction, offers high reproducibility in a short time with simplified manipulation, reduced solvent consumption and lower energy. The achieved results have shown how ultrasound-assisted extraction resulted in shorter extraction time [94,95].

Ethanol extraction has been described as the most suitable medium for the extraction of biologically active phenolic components from propolis [96–99]. In addition, the industrial extraction method commonly used to obtain biocompounds from propolis is conventional extraction (ethanolic or hydroethanolic extraction), where the sample can be submerged in a solvent for days, weeks, or months, which requires an enormous amount of time when extracting on an industrial scale (usually at room temperature) [1,100,101]. Thus, the findings of this study show that the use of ultrasound technology as a treatment during the extraction process is a viable alternative for obtaining antioxidant compounds from propolis in a short period of time when compared with the applied conventional extraction (ethanolic extraction for 7 days) that is usually employed by industry. Furthermore, the ultrasound-assisted method can be efficiently applied to reduce extraction time and energy consumption which is reflected in the lowering of the final cost.

It is important to emphasize that there is little literature on the application of ultrasound technology for obtaining propolis extracts, despite the advantages already mentioned in different studies using other types of matrices [59,68,102–104]. In the study by Taddeo et al. [53], a higher (28% higher) amount of biocompounds was obtained in Italian propolis extracts when using ultrasound exposure combined with conventional solvent extraction. Therefore, the application of ultrasound technology may be useful to increase the extraction of antioxidant compounds in propolis samples when applied in conjunction with the conventional method (ethanolic extraction), or it may reduce extraction time, as shown in this study. Ultrasound-assisted extraction has been confirmed as one of the most economic and efficient extraction methods for recovery of valuable compounds, especially for extraction purposes [94,95].

### Quantification of compounds by HPLC

Analysis by HPLC is an important and efficient technique for the identification of compounds in complex mixtures such as propolis [97,105,106] and enables the quantification of compounds of chemical and biotechnological interest. As previously reported and evidenced in different studies, the chemical composition of propolis depends on its geographical location, and as such, its biological activity is closely related to the native vegetation of the collection site [19,27,107,108]. However, Brazilian red propolis presents a composition similar to that of the Cuban red propolis produced in the province of Pinar Del Rio, without benzophenones, but with several isoflavones, such as medicarpin, homopterocarpin, and formononetin [11,18,109].

Previous studies have shown that formononetin is one of the main components, is an important marker of Brazilian red propolis [32,110,111], and is also present in its botanical origin, *Dalbergia ecastaphyllum (L)Taub* [10,112]. Cavendish et al. [32] demonstrated some biological activities of the hydroalcoholic extract of the red propolis due to the presence of formononetin, as it was antinociceptive and anti-inflammatory in experimental models. Formononetin has also been associated with the reduced action of IL-1β and nuclear factor κB (NF-κB) *in vitro* [113]. In addition, the anti-inflammatory and antioxidant activities of formononetin promoted neural and pulmonary protective effects *in vivo*, decreasing TNF-α and IL-6 levels [114,115] and improving the activity of superoxidase dismutase [116]. These studies evidenced the importance of formononetin in red propolis extracts; therefore, the identification of an efficient method to obtain this important compound is of great relevance.

In the current study, of the ten phenolic compounds investigated (Table 2), only formononetin and kaempferol were found to be above the limits of detection and quantification in the extracts. Quercetin and hydrate rutin were present in the chromatograms obtained, however, below the limits of quantification or detection (S1 Table). Neves et al. [23] investigated ethanol extracts of Brazilian red propolis (two samples from Pernambuco) and found formononetin as the main component (2.86 and 1.71 μg.mg^-1^). The isoflavones rutin (0.21 and 0.02 μg.mg^-1^) and quercetin (0.37 and 0.39 μg.mg^-1^) were present at very low concentrations. Ruffato et al. [117] investigated fractions ethanol extract of the red propolis from Brazil (Alagoas) and also determined formononetin as one of the main biomarkers, in addition to flavonoids biochanin A and liquiritigenin. Similar results have also been demonstrated by Ruffato et al. [6].

**Fig 3:**
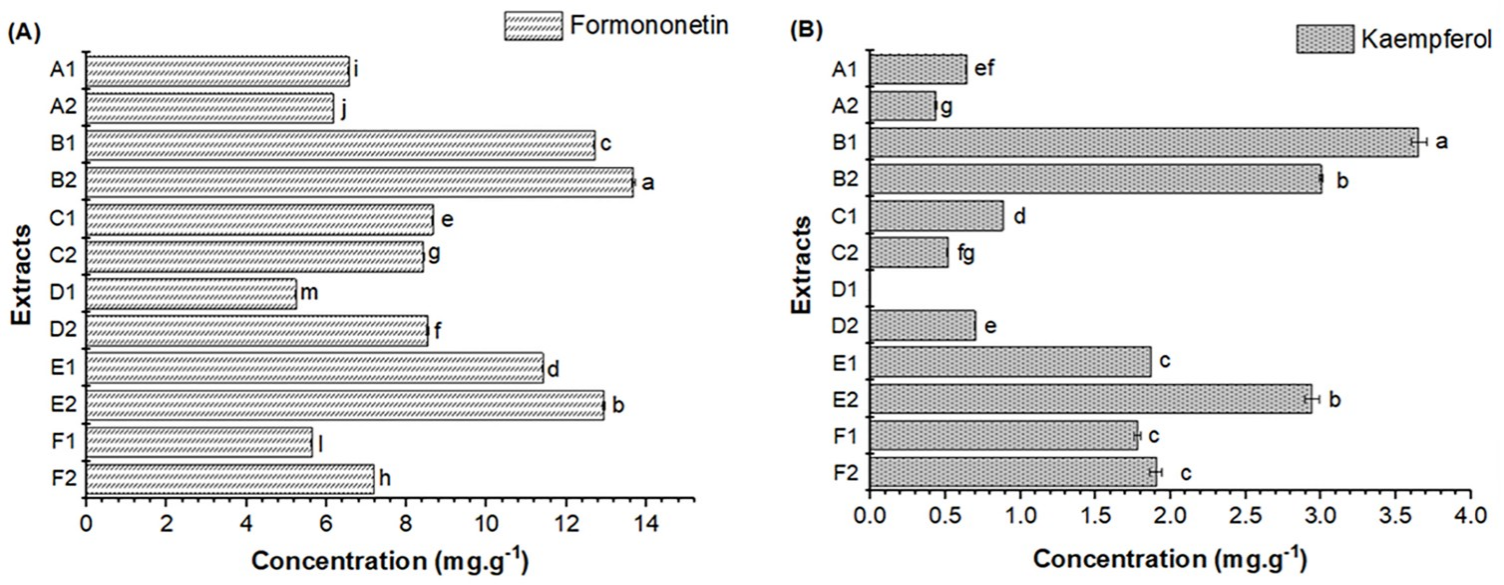
Concentration of formononetin (A) and kaempferol (B) in Brazilian red propolis extracts from different geographical sources obtained by conventional extraction (1) and ultrasound-assisted extraction (2) (mean ± standard error of mean). A1, B1, C1, D1, E1 and F1—Extracts obtained by conventional extraction; A2, B2, C2, D2, E2 and F2—Extracts obtained by ultrasound-assisted extraction. Statistical analysis: Values showing the same letter in the same analysis do not show significant difference (p>0.05) through the Tukey test at a 95% confidence level.

**Fig 4:**
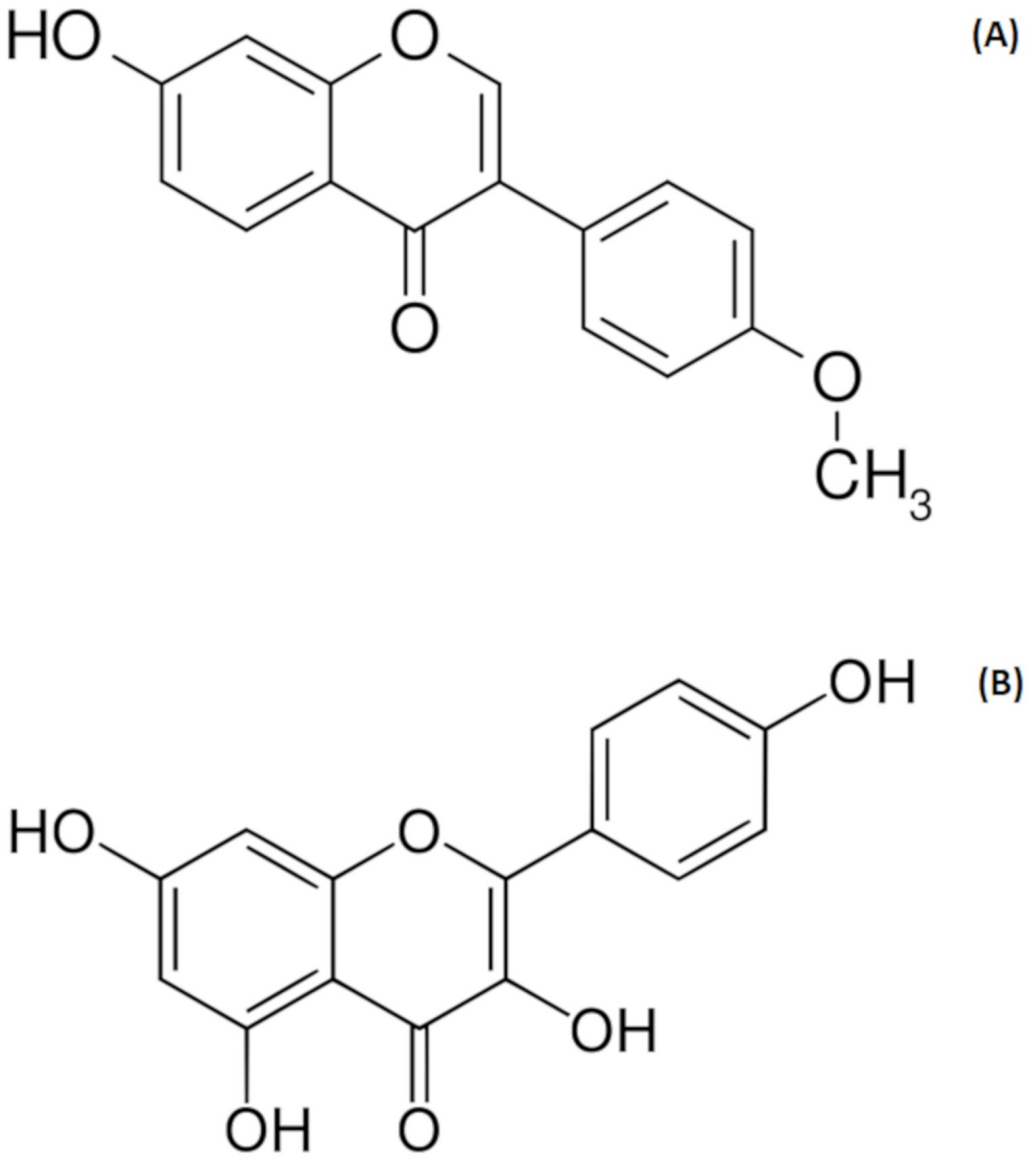
Chemical structures of the biomarkers formononetin (A) and kaempferol (B).

The results of the quantitative analysis of formononetin and kaempferol in the extracts obtained by the two extraction methods from different Brazilian red propolis samples are presented in Table 4 and Fig 3. The chemical structures of the biomarkers formononetin and kaempferol are shown in Fig 4. The formononetin content ranged from 5.22±0.01 (D1) to 13.64±0.04 mg.g^-1^ (B2), whereas the kaempferol content ranged from 0.43±0.01 (A2) to 3.72±0.05 mg.g^-1^ (B1) among the extracts.

**Table 4:** Content of formononetin and kaempferol of extracts from different samples of Brazilian red propolis obtained by conventional (1) and ultrasound-assisted extraction (2) (mean ± standard error of mean).

Sample	Formononetin (mg.g^-1^)	Kaempferol (mg.g^-1^)
A1	6.54±0.01^i^	0.65±0.01^e.f^
A2	6.15±0.01^j^	0.43±0.01^g^
B1	12.67±0.01^c^	3.72±0.05^a^
B2	13.64±0.04^a^	3.02±0.01^b^
C1	8.68±0.01^e^	0.88±0.00^d^
C2	8.40±0.01^g^	0.51±0.00^f.g^
D1	5.22±0.01^m^	---------
D2	8.49±0.02^f^	0.69±0.00^e^
E1	11.39±0.01^d^	1.87±0.00^c^
E2	12.88±0.03^b^	2.94±0.05^b^
F1	5.63±0.01^l^	1.76±0.02^c^
F2	7.17±0.01^h^	1.95±0.04^c^

The results of the quantitative analysis of formononetin and kaempferol in the extracts obtained by the two extraction methods from different Brazilian red propolis samples are presented. The samples A1, B1, C1, D1, E1 and F1 are the extracts obtained by conventional extraction and the samples A2, B2, C2, D2, E2 and F2 are the extracts obtained by ultrasound-assisted extraction.

Statistical analysis: Values showing the same letter in the same column do not show significant difference (p>0.05) through the Tukey test at a 95% confidence level.

The extracts from sample B (Alagoas) presented the highest contents of the analyzed compounds, being the sample that also exhibited the highest content of total phenolic compounds (Fig 2 and Table 3). As expected, formononetin was present in significant amounts in all extracts, regardless of the geographical origin of the sample or the extraction method employed. Lopez et al. [11] investigated red propolis samples of different origins to identify the main chemical markers by mass spectrometry. In that study, formononetin was present at significant concentrations in 10 of the 14 investigated samples and was considered as the main marker of this type of propolis.

According to the results in Table 4 and Fig 3, significant differences (p>0.05) were observed for the levels of formononetin and kaempferol when comparing the two extraction methods applied for the same sample, and when comparing the extracts obtained by the same method for samples of different origins. Thus, this study also proves that the extraction method [50,118–120] and geographical origin [121,122] influence the content of specific compounds in propolis extracts. The achieved results and statistical analysis have shown how ultrasound-assisted extraction resulted in shorter extraction time, and increased extraction capacity of biomarkers with high antioxidant activity from Brazilian red propolis.

In this study, ethanol extraction combined with ultrasound was more efficient for extracting the formononetin compound (Table 4 and Fig 3) (p>0.05). Thus, the ultrasound application was extremely efficient for the extraction of formononetin from extracts of red propolis. Ultrasound has been applied in different studies for intensification of bioactive compounds extraction [123–125].

For the other investigated compound, kaempferol, it was not possible to determine which method was the most efficient, since there was a variation depending on the sample analyzed. For example, for samples A, B, and C, conventional extraction was superior, whereas for samples D and E, the application of ultrasound had a very significant effect on kaempferol extraction (p>0.05). However, from the results found in this study, it can be stated that the application of ultrasound is efficient to obtain extracts with high content of antioxidant compounds, such as the formononetin and kaempferol, and as a faster extractive method, compared to conventional extraction.

Andrade et al. [66], Machado et al. [27], Szliszka et al. [126], and Jansen-Alves et al. [127] found kaempferol in samples of Brazilian green propolis, and it was considered to be one of the main constituents of this type of propolis. In the current study, significant amounts of kaempferol were identified in the red propolis extracts from northeastern Brazil (Fig 3). These results may suggest that other plant species [11,18,128,129] in addition to *Dalbergia ecastaphyllum (L)Taub* are important sources of resins for red propolis in northeastern Brazil. Similar results were obtained by Andrade et al. [65] and Andrade et al. [66], who identified the presence of kaempferol in ethanol extracts of red propolis from the Brazilian states of Sergipe and Alagoas, respectively.

Important biological effects have been reported for kaempferol in recent studies [127,130,131]. In addition to propolis, kaempferol is a flavonoid found in botanical products that are commonly used in traditional medicines, such as *Ginkgo biloba* [132,133] and *Sophora japonica* [134–136]. Kaempferol and some of its glycosides have different pharmacological activities, including antioxidant, anti-inflammatory, anti-cancer, anti-diabetic, and anti-osteoarthritis activity [137–140].

Filomeni et al. [141] demonstrated the neuroprotective effect of kaempferol on SH-SY5Y cells and primary neurons from rotenone toxicity, such as a reduction in caspase cleavage and apoptotic nuclei. Kaempferol has also been associated with a protective effect in the brains of rats with induced ischemic injury [142].

As showed in this study, ultrasound-assisted extraction can also provide the opportunity for enhanced extraction of specific bioactive components at lower processing time [143], and is more effective than conventional ethanolic extraction for obtaining many compounds from natural matrices using between 15–60 minutes of extraction [93,144].

Based on the results of the chromatographic analysis, red propolis extracts from northeastern Brazil obtained by ultrasound-assisted extraction are important sources of formononetin and kaempferol, which are described in the literature as having a high biotechnological potential given their demonstrated pharmacological effects. Therefore, considering the antioxidant potential of the extracts, they can be considered as important candidates for use in new functional foods or new drugs.

### Determination of antitumoral activity *in vitro*

**Fig 5:**
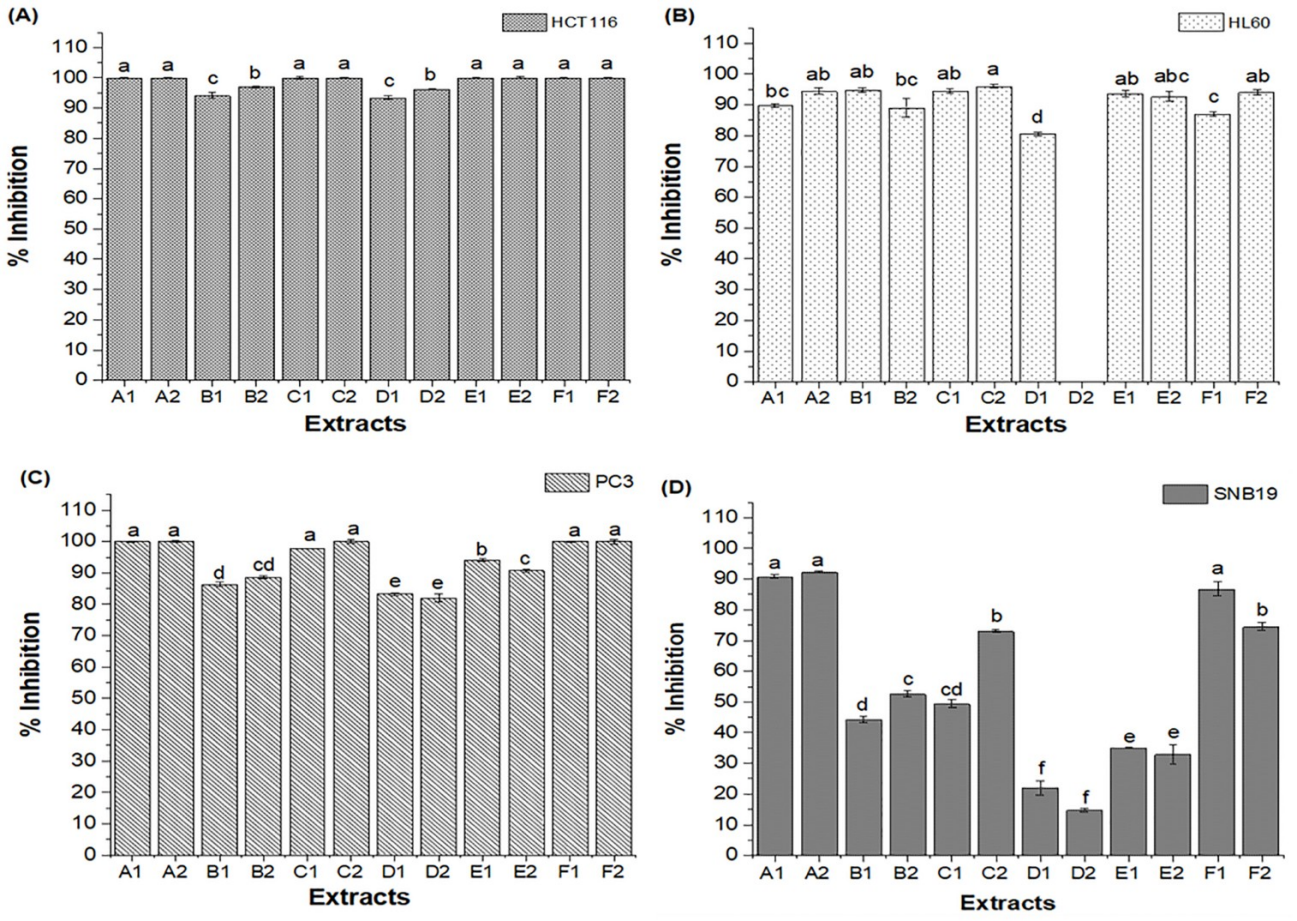
Percentage growth inhibition of tumor cell lines by propolis extracts obtained by conventional extraction (1) and ultrasound-assisted extraction (2): (A) HCT-116 (colon carcinoma), (B) HL-60 (leukemia), (C) PC3 (prostate carcinoma), and (D) SNB19 (glioblastoma). A1, B1, C1, D1, E1 and F1—Extracts obtained by conventional extraction; A2, B2, C2, D2, E2 and F2—Extracts obtained by ultrasound-assisted extraction. Statistical analysis: Values showing the same letter for the same analysis do not show significant differences (p>0.05) through the Tukey test at a 95% confidence level.

The present study also investigated the cytotoxicity of the extracts from the six red propolis samples obtained by conventional extraction and ultrasound-assisted extraction against four tumor cell lines: HCT116 (human colon), HL60 (leukemia), PC3 (prostate carcinoma), and SNB19 (glioblastoma), with the aim of evaluating antiproliferative effects, as shown in Fig 5 (percentage inhibition).

Fig 5 shows that red propolis extracts (tested at a concentration of 50 μg.mL^-1^) altered the viability of the investigated cell lines (Fig 5A–5D), with a significant reduction (p<0.05) at the final cell concentration (except for the D2 extract against the HL60 line). Franchi et al. [145] comparatively evaluated propolis extracts of different types and geographical origins and found a higher antiproliferative activity for the extracts obtained from the red propolis samples, evidencing the high biological potential of this matrix due mainly to its differentiated composition. Machado et al. [146] evaluated the chemical composition and biological activity of yellow, green, brown, and red Brazilian propolis and found the highest selectivity against all tumor cells was shown by red propolis especially against HL60.

Awale et al. [29] found similar cytotoxic effects when comparing Brazilian red propolis extracts and antitumor drugs, such as 5-fluorouracil and doxorubicin, in six tumor cell lines (including HCT-116), thus evidencing the biological potential of this natural matrix. Frozza et al. [91] also showed the *in vitro* antiproliferative effect of Brazilian red propolis against human laryngeal squamous cell carcinoma (Hep-2), human cervical adenocarcinoma cells (HeLa), and normal human embryonic kidney cells (Hek-293).

For the HCT-116 cell line (colon carcinoma) (Fig 5A), regardless of the geographical origin of the sample or the extraction conditions employed, all extracts had a percent inhibition greater than 90%. In general, few significant differences (p>0.05) were observed for the percent inhibition for the HL-60 (leukemia) (Fig 5B) and PC3 (prostate carcinoma) cell lines (Fig 5C) when evaluating the different extracts (two extraction methods and six samples from different sources). For these lines, all of the extracts had a percent inhibition above 80% (except for extract D2, which showed no inhibition against HL-60). In general, for these three tumor lines, the extraction method used did not influence the cytotoxic response (Fig 5A, 5B and 5C).

However, significant differences (p>0.05) in percent inhibition were observed between the extracts against SNB19 (glioblastoma) (Fig 5D), and thus, the cytotoxic response was related to the origin of the sample. Overall, a lower percent inhibition was obtained against this line, with only four of the twelve evaluated extracts showing a percent inhibition greater than 80%. In addition, it was found that the geographical origin of the sample significantly influenced (p>0.05) the inhibition potential of the SNB19 line (Fig 5D). The extracts obtained from samples A (Alagoas) and F (Sergipe) showed the best results for the antiproliferative activity against SNB19, and these extracts were the only ones that were able to inhibit more than 80% of cell growth for all cancer cell lines investigated in this study. However, comparing the extracts from the same sample (collection source) obtained by the two different methods, in general, no influence of the method on the cytotoxic response was also observed for the SNB19 cell line, as shown in Fig 5D (with the exception of samples B2 and C2 –extracts obtained by ultrasound-assisted extraction were more efficient).

Thus, the results found in this study suggest that in some cases the extraction with ultrasound can positively influence the biological activity against the tumor lines tested. In addition, because it is a faster extraction method, when compared to conventional ethanol extraction, it may be considered as the method of choice to obtain extracts of red propolis from northeastern Brazil (regardless of geographic origin).

Mendonça et al. [147] and Silva et al. [1] also investigated cytotoxic activity against different cell lines (including HCT-116 and SF295) for red propolis extracts from northeastern Brazil and identified the cell proliferation inhibition potential of the extracts. Banzato et al. [148] also found cytotoxic activity of the crude extract and fractions of Brazilian red propolis against seven tumor cell lines that included PC3 (prostate), OVCAR-3 (ovary), K-562 (leukemia), and U251 (glioma). Brazilian red propolis induced cell death and decreased the migration potential of bladder cancer cells, suggesting a potential source for the development of new drugs and/or herbal medicines for the treatment of this type of cancer [149]. Thus, red propolis extracts present high levels of cytotoxicity against different tumor cell lines, as was previously demonstrated in other studies [91,128,150–152].

Although different studies have shown the potent antiproliferative effect of formononetin [114,115,153] and kaempferol [154–156], in this study, it was not possible to establish a direct correlation of the effect of these components on the efficiency of the growth inhibition of the tested cell lines, considering the high percentage inhibition exhibited by the extracts, regardless of the concentration of these compounds (Fig 3 and Table 4).

These results may indicate that the presence of 5.22±0.02 mg.g^-1^ and 0.43±0.01 mg.g^-1^ of formononetin and kaempferol in the extracts, respectively, can be sufficient to achieve a percent inhibition greater than 80% (HCT-116, HL-60, and PC3 lines). In addition, another indicator would be the synergistic action of other phenolic compounds [128,131,157], which were not evaluated in the study but would be present in significant concentrations in the extracts based on the complexity of the chromatograms obtained (S1 Fig). The chemical nature of phenolic compounds and, perhaps, the presence of other compounds contribute to the cytotoxic capacity of the extracts [51]. In addition to formononetin and kaempferol (studied and identified—Tables 2 and 4), biochanin A [18,91,158], daidzein [90] and xanthochymol [159]may be some of the compounds present in extracts of red propolis with synergistic action on cytotoxic activity. According to Hernandez et al. [160], studies investigating the chemical composition of propolis samples can help establishing criteria for the quality control of this matrix, mainly due to its use worldwide and demonstrated differences in relation to geographic origin and extraction method.

For example, the cytotoxic activity of eleven different flavonoids isolated from propolis against colon cancer (HCT-116) and breast cancer (MDA-MB-231) cell lines were investigated by Vukovic et al. [131], who found six flavonoids with potential cytotoxic effects. In the study by Li et al. [128], the cytotoxic activity of 42 compounds isolated from red propolis against six different tumor cell lines was investigated. Although formononetin showed good results, the authors found that the compounds (2S)-7-hydroxy-6-methoxyflavanone and (3S)-mucronulatol presented the best antiproliferative effects against the studied lines (26-L5, B16-BL6, LLC, A549, HeLa, HT-1080), suggesting that these flavonoids could be good candidates for the development of anticancer drugs.

Based on the results found in this study and the findings in the literature, ethanol extracts of red propolis from northeastern Brazil (treated or not with ultrasound) present high antiproliferative capacities against different tumor cell lines. However, the application of ultrasound was efficient for obtaining red propolis extracts in a shorter time when compared to the conventional method and resulted in extracts with important cytotoxic effects *in vitro*. Because of this, we suggested that assisted-ultrasound extraction may be considered as a more efficient technology for the extraction of red propolis from northeastern Brazil. Future studies are needed to demonstrate the safety of using red propolis extracts *in vivo* [161], given its wide application in food, pharmaceuticals, and cosmetics.

## Conclusions

In this study, the levels of flavonoids, phenolic compounds, antioxidant activity, and cytotoxicity against different tumor cell lines were determined for red propolis extracts from different geographical origins and obtained by two extraction methods. The results showed an effect of the origin and of the extraction method in the chemical profile and biological activity of these extracts. We suggested that propolis extracts showed a high in vitro antioxidant activity. The application of ultrasound technology to obtain extracts rich in active compounds proved to be efficient, mainly due to the shorter time needed to obtain the extracts, thus enabling production on an industrial scale.

Therefore, our results demonstrated that extracts from Brazilian red propolis obtained by conventional extraction or assisted-ultrasound extraction may act in a selective way against tumor cells and show potential antitumor activity. Propolis has been a subject of intensive research, especially in the area of cancer. Future studies are needed to evaluate the biological potential of these extracts with *in vivo* models.

## Supporting information

S1 Fig Chromatogram obtained from extract C1 (sample of red propolis from Bahia). (DOCX)

S1 Table Evaluation by HPLC-DAD of the biochemical content of extracts of red propolis obtained by conventional (1) and ultrasound-assisted extraction (2) (mean ± standard error of mean). (DOCX)

S2 Table Raw data from formonometin analysis (HPLC) (mean ± standard deviation). (DOCX)

S3 Table Raw data from kaempferol analysis (HPLC) (mean ± standard deviation). (DOCX)

S4 Table Standards and parameters used for the analysis of the phenolic compounds in the different extracts by HPLC. (DOCX)

S5 Table Raw data from the analysis of antioxidant compounds phenolic compounds (mgGAE.g^-1^), flavonoids (mgQE.g^-1^) and DPPH (IC50) (μg.mL^-1^) (mean ± standard derivation). (DOCX)

S6 Table Raw data from the cytotoxic analysis (mean ± standard deviation). (DOCX)
