# Supplementary material for: Evaluation of the antioxidant profile and cytotoxic activity of red propolis extracts from different regions of northeastern Brazil obtained by conventional and ultrasound-assisted extraction
Source: PLoS One. 2019 Jul 5;14(7):e0219063. doi: 10.1371/journal.pone.0219063 (PMC6611595; doi:10.1371/journal.pone.0219063)
Supplement: S1 Fig — (DOCX) [file pone.0219063.s001.docx]

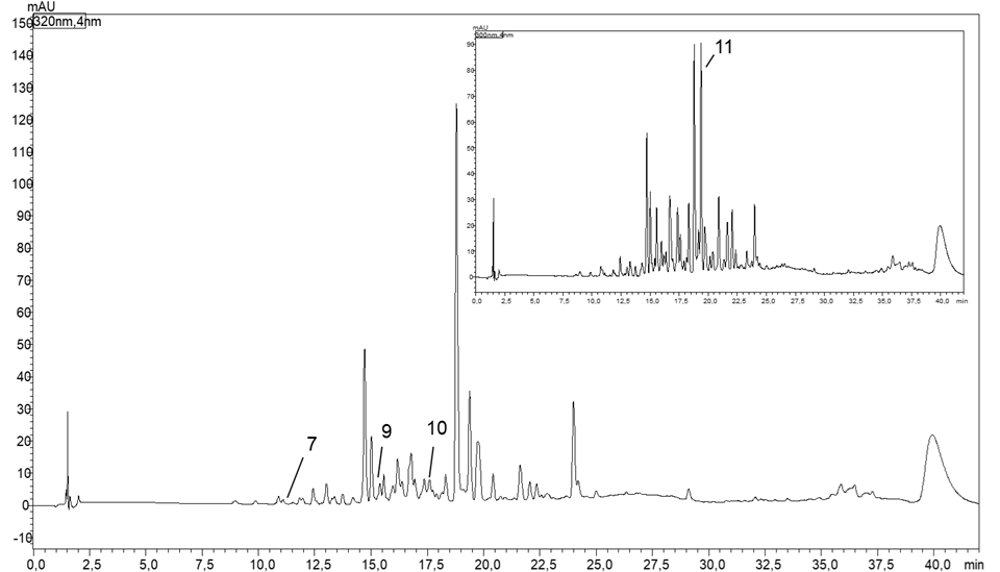


**S1 Fig.** Chromatogram obtained from extract C1 (Sample of red propolis from Bahia). 7 – Rutin Hydrate; 9 – Quercetin; 10 – Kaempferol; and 11 – Formononetin.
