# Supplementary material for: Evaluation of the antioxidant profile and cytotoxic activity of red propolis extracts from different regions of northeastern Brazil obtained by conventional and ultrasound-assisted extraction
Source: PLoS One. 2019 Jul 5;14(7):e0219063. doi: 10.1371/journal.pone.0219063 (PMC6611595; doi:10.1371/journal.pone.0219063)
Supplement: S1 Table — (DOCX) [file pone.0219063.s002.docx]

**S1 Table.** Evaluation by HPLC-DAD of the biochemical content of extracts of red propolis obtained by conventional (1) and ultrasound-assisted extraction (2) (mean ± standard error of mean).

| **Standard**  **(mg.g^-1^)** | **Extracts** | | | | | | | | | | | |
| --- | --- | --- | --- | --- | --- | --- | --- | --- | --- | --- | --- | --- |
|  | **A1** | **A2** | **B1** | **B2** | **C1** | **C2** | **D1** | **D2** | **E1** | **E2** | **F1** | **F2** |
| **Formonometin** | 6.54±0.01^i^ | 6.15±0.01^j^ | 12.67±0.01^c^ | 13.64±0.04^a^ | 8.68±0.01^e^ | 8.40±0.01^g^ | 5.22±0.01^m^ | 8.49±0.02^f^ | 11.39±0.01^d^ | 12.88±0.03^b^ | 5.63±0.01^l^ | 7.17±0.01^h^ |
| **Kaempferol** | 0.65±0.01^e.f^ | 0.43±0.01^g^ | 3.72±0.05^a^ | 3.02±0.01^b^ | 0.88±0.00^d^ | 0.51±0.00^f.g^ | <LQ | 0.69±0.00^e^ | 1.87±0.00^c^ | 2.94±0.05^b^ | 1.76±0.02^c^ | 1.95±0.04^c^ |
| **Caffeic Acid** | ND | ND | ND | ND | ND | ND | <LD | ND | ND | ND | ND | ND |
| **Gallic acid** | ND | ND | ND | ND | ND | ND | ND | ND | ND | ND | ND | ND |
| ***p*-coumaric acid** | ND | <LD | ND | <LD | ND | <LD | ND | ND | ND | <LD | ND | ND |
| **Acid**  **Trans-Ferulic** | ND | ND | ND | ND | ND | ND | ND | ND | ND | ND | ND | ND |
| **Catequin** | ND | ND | ND | ND | ND | ND | ND | ND | ND | ND | ND | ND |
| **Epicatequin** | ND | ND | ND | ND | ND | ND | ND | ND | ND | ND | ND | ND |
| ***O*-dianisidine** | ND | ND | ND | ND | ND | ND | ND | ND | ND | ND | ND | ND |
| **Quercetin** | <LQ | <LD | <LQ | <LQ | <LQ | <LQ | <LQ | <LQ | ND | <LQ | <LQ | ND |
| **Hydrate rutin** | <LD | <LD | <LD | <LD | <LD | <LD | <LQ | <LD | <LD | <LQ | <LD | <LD |

ND=not detected; <LQ=low limit of quantification; <LD=low limit of detection.

A1, B1, C1, D1, E1 and F1 – Extracts obtained by conventional extraction; A2, B2, C2, D2, E2 and F2 – Extracts obtained by ultrasound-assisted extraction.

Statistical analysis: Values showing the same letter in the same column do not show significant difference (p>0.05) through the Tukey test at a 95% confidence level.
