# Supplementary material for: Evaluation of the antioxidant profile and cytotoxic activity of red propolis extracts from different regions of northeastern Brazil obtained by conventional and ultrasound-assisted extraction
Source: PLoS One. 2019 Jul 5;14(7):e0219063. doi: 10.1371/journal.pone.0219063 (PMC6611595; doi:10.1371/journal.pone.0219063)
Supplement: S2 Table — (DOCX) [file pone.0219063.s003.docx]

**S2 Table. Raw data from formonometin analysis (HPLC) (mean ± standard deviation).**

| **Sample** | **Formonometin** | | | |
| --- | --- | --- | --- | --- |
|  | **Concentration** | **Mean** | **standard deviation (SD)** | **Coefficient of Variation (CV) %** |
| A1 | 6.5419 | 6.54 | 0.02 | 0.26 |
|  | 6.5755 |  |  |  |
|  | 6.5528 |  |  |  |
| A2 | 6.1509 | 6.15 | 0.01 | 0.22 |
|  | 6.1769 |  |  |  |
|  | 6.1713 |  |  |  |
| B1 | 12.6749 | 12.67 | 0.03 | 0.20 |
|  | 12.7060 |  |  |  |
|  | 12.7262 |  |  |  |
| B2 | 13.6380 | 13.64 | 0.07 | 0.51 |
|  | 13.6300 |  |  |  |
|  | 13.7542 |  |  |  |
| C1 | 8.6765 | 8.68 | 0.02 | 0.25 |
|  | 8.6332 |  |  |  |
|  | 8.6561 |  |  |  |
| C2 | 8.4036 | 8.40 | 0.02 | 0.23 |
|  | 8.4427 |  |  |  |
|  | 8.4202 |  |  |  |
| D1 | 5.2190 | 5.22 | 0.02 | 0.40 |
|  | 5.2110 |  |  |  |
|  | 5.2506 |  |  |  |
| D2 | 8.4940 | 8.49 | 0.03 | 0.36 |
|  | 8.5443 |  |  |  |
|  | 8.5498 |  |  |  |
| E1 | 11.3864 | 11.39 | 0.02 | 0.20 |
|  | 11.4168 |  |  |  |
|  | 11.4301 |  |  |  |
| E2 | 12.8818 | 12.88 | 0.05 | 0.42 |
|  | 12.9212 |  |  |  |
|  | 12.9896 |  |  |  |
| F1 | 5.6322 | 5.63 | 0.03 | 0.45 |
|  | 5.5886 |  |  |  |
|  | 5.6333 |  |  |  |
| F2 | 7.1738 | 7.17 | 0.01 | 0.14 |
|  | 7.1926 |  |  |  |
|  | 7.1790 |  |  |  |
