# Supplementary material for: Evaluation of the antioxidant profile and cytotoxic activity of red propolis extracts from different regions of northeastern Brazil obtained by conventional and ultrasound-assisted extraction
Source: PLoS One. 2019 Jul 5;14(7):e0219063. doi: 10.1371/journal.pone.0219063 (PMC6611595; doi:10.1371/journal.pone.0219063)
Supplement: S3 Table — (DOCX) [file pone.0219063.s004.docx]

**S3 Table. Raw data from kaempferol analysis (HPLC) (mean ± standard deviation).**

| **Sample** | **Kaempferol** | | | |
| --- | --- | --- | --- | --- |
|  | **Concentration** | **Mean** | **standard deviation (SD)** | **Coefficient of Variation (CV) %** |
| A1 | 0.6471 | 0.65 | 0.01 | 1.44 |
|  | 0.6308 |  |  |  |
|  | 0.6311 |  |  |  |
| A2 | 0.4290 | 0.43 | 0.01 | 2.75 |
|  | 0.4453 |  |  |  |
|  | 0.4223 |  |  |  |
| B1 | 3.7198 | 3.72 | 0.09 | 2.43 |
|  | 3.6888 |  |  |  |
|  | 3.5498 |  |  |  |
| B2 | 3.0194 | 3.02 | 0.02 | 0.61 |
|  | 3.0101 |  |  |  |
|  | 2.9837 |  |  |  |
| C1 | 0.8834 | 0.88 | 0.00 | 0.14 |
|  | 0.8810 |  |  |  |
|  | 0.8827 |  |  |  |
| C2 | 0.5116 | 0.51 | 0.00 | 0.36 |
|  | 0.5117 |  |  |  |
|  | 0.5148 |  |  |  |
| D1 | 0.0000 | 0.00 | 0.00 | 0.00 |
|  | 0.0000 |  |  |  |
|  | 0.0000 |  |  |  |
| D2 | 0.6940 | 0.69 | 0.00 | 0.35 |
|  | 0.6964 |  |  |  |
|  | 0.6989 |  |  |  |
| E1 | 1.8682 | 1.87 | 0.00 | 0.01 |
|  | 1.8684 |  |  |  |
|  | 1.8687 |  |  |  |
| E2 | 2.9448 | 2.94 | 0.08 | 2.83 |
|  | 3.0212 |  |  |  |
|  | 2.8544 |  |  |  |
| F1 | 1.7588 | 1.76 | 0.04 | 2.07 |
|  | 1.7595 |  |  |  |
|  | 1.8222 |  |  |  |
| F2 | 1.9506 | 1.95 | 0.07 | 3.55 |
|  | 1.9347 |  |  |  |
|  | 1.8236 |  |  |  |
