# Supplementary material for: Evaluation of the antioxidant profile and cytotoxic activity of red propolis extracts from different regions of northeastern Brazil obtained by conventional and ultrasound-assisted extraction
Source: PLoS One. 2019 Jul 5;14(7):e0219063. doi: 10.1371/journal.pone.0219063 (PMC6611595; doi:10.1371/journal.pone.0219063)
Supplement: S4 Table — (DOCX) [file pone.0219063.s005.docx]

**S4 Table. Standards and parameters used for the analysis of the phenolic compounds in the different extracts by HPLC.**

| **Standard** | **t_R_(min.)** | **ʎ (nm)** | **Stock solution (mg L^-1^)** | **Range (mg L-1)** | **Limit of detection (mg L^-1^)** | **Limit of quantification (mg L^-1^)** |
| --- | --- | --- | --- | --- | --- | --- |
| Gallic acid | 2.26 | 280 nm | 540 | 1.0 - 12.5 | 0.92 | 3.05 |
| Caffeic acid | 8.129 | 300 nm | 510 | 1.0 – 15.0 | 0.82 | 2.73 |
| Trans-ferulic acid | 11.375 | 320 nm | 550 | 0.5 - 12.5 | 0.28 | 0.92 |
| p-Coumaric acid | 10.359 | 300 nm | 550 | 1.0 - 15.0 | 0.82 | 2.72 |
| Catechin | 6.423 | 280 nm | 500 | 1.0 – 15.0 | 0.81 | 2.68 |
| Epicatechin | 8.438 | 280 nm | 530 | 0.5 - 15.0 | 0.28 | 0.93 |
| Formononetin | 19.462 | 300 nm | 530 | 0.5 - 12.5 | 0.31 | 1.02 |
| Kaempferol | 17.533 | 320 nm | 340 | 0.5 - 12.5 | 0.12 | 0.41 |
| Quercetin | 15.302 | 320 nm | 545 | 0.5 - 12.5 | 0.21 | 0.71 |
| Rutin hydrate | 11 | 320 nm | 505 | 0.5 - 12.5 | 0.27 | 0.91 |
