# Supplementary material for: Evaluation of the antioxidant profile and cytotoxic activity of red propolis extracts from different regions of northeastern Brazil obtained by conventional and ultrasound-assisted extraction
Source: PLoS One. 2019 Jul 5;14(7):e0219063. doi: 10.1371/journal.pone.0219063 (PMC6611595; doi:10.1371/journal.pone.0219063)
Supplement: S5 Table — (DOCX) [file pone.0219063.s006.docx]

**S5 Table. Raw data from the analysis of antioxidant compounds Phenolic compounds (mgGAE.g^-1^), Flavonoids (mgQE.g^-1^) and DPPH (IC_50_) (µg.mL^-1^) (mean ± standard deviation)**

| Samples | Phenolic compounds (mgGAE.g^-1^) | Flavonoids (mgQE.g^-1^) | DPPH (IC_50_) (µg.mL^-1^) |
| --- | --- | --- | --- |
| A1 | 307.63±0.92 | 81.42±4.45 | 57.27±0.73 |
| A2 | 337.72±13.08 | 108.02±0.18 | 48.00±2.45 |
| B1 | 398.31±11.15 | 62.01±0.51 | 70.41±3.22 |
| B2 | 380.73±13.60 | 61.17±1.18 | 72.02±2.79 |
| C1 | 308.49±6.91 | 82.87±0.35 | 76.58±4.17 |
| C2 | 314.75±14.00 | 90.38±3.36 | 72.70±3.01 |
| D1 | 277.81±1.32 | 57.07±2.20 | 103.85±1.23 |
| D2 | 283.74±5.17 | 65.34±0.85 | 94.28±1.82 |
| E1 | 332.74±11.68 | 42.00±0.75 | 102.94±5.94 |
| E2 | 335.16±12.55 | 43.64±1.90 | 90.61±2.98 |
| F1 | 333.06±9.39 | 75.89±2.50 | 65.96±0.10 |
| F2 | 334.89±15.34 | 79.67±2.10 | 47.42±4.28 |
