# Supplementary material for: Evaluation of the antioxidant profile and cytotoxic activity of red propolis extracts from different regions of northeastern Brazil obtained by conventional and ultrasound-assisted extraction
Source: PLoS One. 2019 Jul 5;14(7):e0219063. doi: 10.1371/journal.pone.0219063 (PMC6611595; doi:10.1371/journal.pone.0219063)
Supplement: S6 Table — (DOCX) [file pone.0219063.s007.docx]

**S6 Table. Raw data from the cytotoxic analysis (mean ± standard deviation).**

| Samples | PC3 |  | HCT116 |  | SNB19 |  | HL60 |  |
| --- | --- | --- | --- | --- | --- | --- | --- | --- |
|  | **Mean Inhibition %** | **standard deviation (SD)** | **Mean Inhibition %** | **standard deviation (SD)** | **Mean** | **standard deviation (SD)** | **Mean** | **standard deviation (SD)** |
| A1 | 100 | 0.33 | 100 | 0.05 | 90.83 | 0.96 | 89.75 | 1.18 |
|  |  |  |  |  |  |  |  |  |
|  |  |  |  |  |  |  |  |  |
| A2 | 100 | 0.62 | 100 | 0.09 | 92.4 | 0.61 | 94.43 | 1.74 |
|  |  |  |  |  |  |  |  |  |
|  |  |  |  |  |  |  |  |  |
| B1 | 86.28 | 1.18 | 94.09 | 1.75 | 44.28 | 1.55 | 94.83 | 1.31 |
|  |  |  |  |  |  |  |  |  |
|  |  |  |  |  |  |  |  |  |
| B2 | 88.62 | 0.96 | 97 | 0.51 | 52.61 | 1.69 | 89.08 | 5.27 |
|  |  |  |  |  |  |  |  |  |
|  |  |  |  |  |  |  |  |  |
| C1 | 97.73 | 0.11 | 100 | 0.66 | 49.41 | 2.25 | 94.55 | 1.25 |
|  |  |  |  |  |  |  |  |  |
|  |  |  |  |  |  |  |  |  |
| C2 | 100 | 1.16 | 100 | 0.16 | 73.1 | 0.87 | 96.05 | 1.15 |
|  |  |  |  |  |  |  |  |  |
|  |  |  |  |  |  |  |  |  |
| D1 | 83.26 | 0.8 | 93.42 | 0.94 | 21.94 | 3.81 | 80.59 | 0.95 |
|  |  |  |  |  |  |  |  |  |
|  |  |  |  |  |  |  |  |  |
| D2 | 81.94 | 2.15 | 96.2 | 0.14 | 14.74 | 0.94 | 0 | 0 |
|  |  |  |  |  |  |  |  |  |
|  |  |  |  |  |  |  |  |  |
| E1 | 94.07 | 0.69 | 100 | 0.14 | 35 | 0.17 | 93.65 | 1.6 |
|  |  |  |  |  |  |  |  |  |
|  |  |  |  |  |  |  |  |  |
| E2 | 90.7 | 0.79 | 100 | 0.51 | 32.92 | 5.46 | 92.68 | 2.82 |
|  |  |  |  |  |  |  |  |  |
|  |  |  |  |  |  |  |  |  |
| F1 | 100 | 0.26 | 100 | 0.23 | 86.77 | 3.97 | 87.02 | 1.22 |
|  |  |  |  |  |  |  |  |  |
|  |  |  |  |  |  |  |  |  |
| F2 | 100 | 1.19 | 100 | 0.05 | 74.56 | 2.34 | 94.1 | 1.4 |
|  |  |  |  |  |  |  |  |  |
|  |  |  |  |  |  |  |  |  |
